# Antibiotic Resistance Patterns in Invasive Group B Streptococcal Isolates

**DOI:** 10.1155/2008/727505

**Published:** 2009-02-05

**Authors:** Mei L. Castor, Cynthia G. Whitney, Kathryn Como-Sabetti, Richard R. Facklam, Patricia Ferrieri, Joanne M. Bartkus, Billie A. Juni, Paul R. Cieslak, Monica M. Farley, Nellie B. Dumas, Stephanie J. Schrag, Ruth Lynfield

**Affiliations:** ^1^Infectious Disease Epidemiology, Prevention and Control Division, Minnesota Department of Health, 625 Robert Street North, P.O. Box 64975, Saint Paul, MN 55164, USA; ^2^Epidemic Intelligence Service, Epidemiology Program Office, Centers for Disease Control and Prevention, 1600 Clifton Road NE, Atlanta, GA 30333, USA; ^3^Respiratory Diseases Branch, Division of Bacterial and Mycotic Diseases, National Center for Infectious Diseases, Centers for Disease Control and Prevention, 1600 Clifton Road NE, Atlanta, GA 30333, USA; ^4^Streptococcus Laboratory, Respiratory Diseases Branch, Division of Bacterial and Mycotic Diseases, National Center for Infectious Diseases, Centers for Disease Control and Prevention, 1600 Clifton Road NE, Atlanta, GA 30333, USA; ^5^Pediatrics and Laboratory Medicine and Pathology, Clinical Microbiology Laboratory, University of Minnesota Medical School, 420 Delaware Street SE Minneapolis, MN 55455, USA; ^6^Public Health Laboratory Division, Minnesota Department of Health, 601 Robert Street North, P.O. Box 64899, Saint Paul, MN 55164, USA; ^7^Public Health Division, Oregon Department of Human Services, 800 NE Oregon Street, Portland, OR 97232, USA; ^8^Atlanta Veterans Affairs Medical Center, Emory University School of Medicine, 1670 Clairmont Rd, Station 151 Decatur, GA 30033, USA; ^9^Wadsworth Center, New York State Department of Health, 120 New Scotland Avenue, Albany, NY 12208, USA

## Abstract

Antibiotics are used for both group B streptococcal (GBS) prevention and treatment. Active population-based surveillance for invasive GBS disease was conducted in four states during 1996–2003. Of 3813 case-isolates, 91.0% (3471) were serotyped, 77.1% (2937) had susceptibility testing, and 46.6% (3471) had both. All were sensitive to penicillin, ampicillin, cefazolin, cefotaxime, and vancomycin. Clindamycin and erythromycin resistance was 12.7% and 25.6%, respectively, and associated with serotype V (*P* < .001). Clindamycin resistance increased from 10.5% to 15.0% (*X* ^2^ for trend 12.70; *P* < .001); inducible clindamycin resistance was associated with the *erm* genotype. Erythromycin resistance increased from 15.8% to 32.8% (*X* ^2^ for trend 55.46; *P* < .001). While GBS remains susceptible to beta-lactams, resistance to alternative agents such as erythromycin and clindamycin is an increasing concern.

## 1. INTRODUCTION

Early-onset group B *Streptococcal* (GBS) infection, occurring in the first 7 days of life, is a leading cause of invasive bacterial infection among newborns and generally results from vertical transmission of GBS from colonized mothers to their infants. Neonatal infection is commonly characterized by bacteremia, pneumonia, and meningitis; mortality from early-onset disease is estimated at 5%. GBS disease among pregnant women manifests as urinary tract infections, bacteremia, chorioamnionitis, endometritis, or septic abortions [1]. Among non-pregnant adults, GBS disease typically occurs among older or immunocompromised persons.

In the 1990s, the incidence of early-onset neonatal GBS disease declined dramatically after widespread implementation of intrapartum antibiotic prophylaxis (IAP). Revisions to IAP guidelines in 2002 included universal GBS screening of pregnant women with vaginal and rectal cultures at 35–37 weeks' gestation, with IAP given to those with positive cultures. Susceptibility testing for erythromycin and clindamycin of GBS isolates is recommended for colonized women who are thought to be at high risk for anaphylaxis to penicillin or ampicillin. Cefazolin is recommended for those who cannot take penicillin but are at low risk for anaphylaxis [2].

Publications from the United States and Canada report clindamycin resistance (3%–21%) and erythromycin resistance (5%–29%) in GBS isolates [3–8]. Alteration of the erythromycin-binding site on the 50S ribosomal RNA subunit, conferred by the *erm* gene, and active efflux of the erythromycin, conferred by the *mef* gene, are the most common macrolide resistance mechanisms in beta-hemolytic streptococci [9]. The *erm* gene confers resistance to macrolides, lincosamides, and streptogramin B-type antibiotics (MLS_B_) and may be expressed constitutively or it may be inducible. The *mef* gene confers resistance only to macrolide antibiotics, the M phenotype. The inducible MLS_B_ phenotype may be detected in the laboratory by a disk approximation method (D-test), which involves placement of an erythromycin and a clindamycin disk in close proximity on the surface of an agar plate. Clinical and Laboratory Standards Institute (CLSI) guidelines for antimicrobial susceptibility testing now include a method for performing the D-test on beta-hemolytic streptococci [10].

Serotype distribution in invasive GBS disease is of interest in determining potential vaccine composition. Serotype III has a high association with late-onset neonatal disease, specifically meningitis. Serotype V has emerged as a more frequent serotype in recent years [11]. Other common serotypes include Ia and II.

We analyzed GBS surveillance data from four distinct US sites to understand serotype and antimicrobial susceptibility patterns in invasive isolates from perinatal (pregnant and neonatal cases) and non-pregnant adult populations.

## 2. METHODS

Invasive GBS isolate data regarding antimicrobial susceptibility, serotype, and epidemiologic data were collected from selected counties in four states (Georgia, Minnesota, New York, and Oregon); all participated in the Centers for Disease Control and Prevention's (CDC) Active Bacterial Core surveillance (ABCs) system of the Emerging Infections Program network for 1996–2003. Georgia's initial surveillance area of eight counties in the Atlanta area expanded in 1997 to 20 counties. New York's initial surveillance area of seven counties in 1997 expanded to 15 counties in 2000 (seven-county Rochester area and eight-county Albany area). Oregon's three-county and Minnesota's statewide surveillance areas remained unchanged throughout the study period. Total surveillance populations ranged from 8.7 million in 1996 to 13.2 million in 2003. By state, 2003 surveillance populations were 4.5 million in Georgia, 5 million in Minnesota, 2.1 million in New York, and 1.5 million in Oregon.

A case of invasive GBS was defined by bacterial isolation from a normally sterile site in a surveillance area resident. GBS isolation from amniotic fluid or placentas was included when fetal demise occurred. Early-onset neonatal cases were classified by a first positive culture on days 0–6 of life and late-onset at 7–89 days of life. Maternal cases were those identified during the antepartum or postpartum periods (30 days after a delivery or miscarriage). The case-finding methodology used for the ABCs system has been previously described [12]. Laboratory audits are performed every 6 months to capture any missed cases. Antimicrobial susceptibility testing was performed at the Minnesota Department of Health (MDH) laboratory or CDC laboratories. Serotyping of Minnesota isolates was performed at the University of Minnesota and CDC, and isolates from other geographic sites were tested at CDC. Lancefield immunoprecipitation techniques were used for serotyping. Antimicrobial susceptibility testing was performed using broth microdilution in microtiter plates containing cation-adjusted Mueller Hinton broth with 3% lysed horse blood (PML Microbiologicals, Wilsonville, Ore) with CLSI susceptibility definitions used when available.

The constitutive (c) MLS_B_ phenotype was inferred from broth microdilution MIC data. Isolates found to be erythromycin-resistant and clindamycin-susceptible were evaluated for inducible (i) clindamycin resistance using the double disk diffusion (D-test) method with clindamycin (2 *μ*g) and erythromycin (15 *μ*g) disks (Becton Dickinson Diagnostic Systems, Sparks, Md) placed 15 mm apart. Strains that remained clindamycin-susceptible (those that did not have flattening of the susceptibility zone adjacent to the erythromycin disk) were determined to be of the M phenotype.

PCR was used to detect the presence of the most common macrolide resistance determinants in all isolates with an M or iMLS_B_ phenotype. Testing was also done on a small subset of isolates with a cMLS_B_ resistance phenotype. PCR for detection of *erm*A, *erm*B, and *erm*C was performed as described by Sutcliffe et al. [13]. Detection of *erm*TR and *mef* (the primers amplify both *mef *A and *mef *E) was performed using primers designed at MDH. The PCR reactions for *erm*TR and *mef *A/E contained 10 mM Tris HCl, 50 mM KCl, 1.5 mM MgCl_2_, 1 *μ*M forward and reverse primers, and 2 *μ*l of template DNA in a total volume of 50 *μ*L. Primers used for the amplification of *erm*TR, a subclass of *erm*A [14], were TR-322U, 5′-GGGTCAGGAAAAGGACAT-3′, and TR619L, 5′-CCTAAAGCTCGTTGGGTATT-3′. Primers used for the amplification of *mef *A/E were mef-3301U, 5′-AGGGCAAGCAGTATCATTAATCA-3′, and mef-3673L, 5′-CTGCAAAGACTGACTATAGCCT-3′. Optimum annealing temperatures for *erm*TR and *mef *PCR were 56°C and 55°C, respectively. PCR products were resolved on a 2% agarose gel. The expected fragment size for *erm*TR was 317 bp and for *mef* was 394 bp.

SAS (version 9.1) was used for frequency and univariate analysis. Trend analyses were performed with Epi Info (CDC, Atlanta, Ga) statistical software (version 6.04c). Chi-square calculations were used to compare continuous variables, with Yates continuity-corrected chi-square used to compare categorical variables. The Mantel-Haenszel chi-square test for trend was used to examine trends across the study period; the Mantel-Haenszel extended test adjusted for possible confounders. An alpha ≤0.05-significance level was used.

## 3. RESULTS

Of the 5 373 patients, 71% had isolates submitted to a public health laboratory and the antimicrobial susceptibility testing was performed on 77.1% (2 937). The plurality of isolates were from Minnesota (46.8% or 1 375), followed by Georgia (26.1% or 768), New York (16.9% or 496), and Oregon (10.2% or 300). The perinatal category comprised 24.4% (1 311) of the total sample with 12.5% (671) early-onset, 7.9% (424) late-onset, and 4.0% (216) maternal patients. The non-pregnant adult category accounted for 73.5% (3 951) of the total.

All 2 937 isolates were susceptible to penicillin, ampicillin, cefotaxime, and vancomycin. Although no CLSI breakpoints existed for the first-generation cephalosporins (cephalothin and cefazolin), all the isolates tested had minimum inhibitory concentrations (MICs) ≤ 0.5 mcg/mL and were, therefore, considered susceptible. Similarly, no breakpoints existed for cefuroxime and cefoxitin. All 1 201 Minnesota isolates tested for cefuroxime susceptibility had MICs ≤ 0.12 mcg/mL and were, therefore, considered susceptible. Of 2 937 isolates tested for cefoxitin susceptibility, 36.7% (1 078) had MICs ≤ 2 mcg/mL; 62.5% (1 835) had MICs = 4 mcg/mL; 0.7% (22) had MICs = 8 mcg/mL; and 0.1% (4) had MICs ≥ 16 mcg/mL.

**Figure 1:**
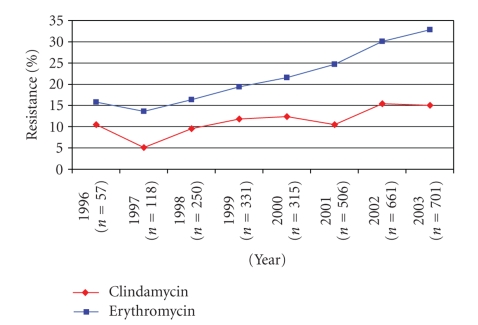
Clindamycin and erythromycin resistance among invasive Group B streptococci isolates by year (*n* = 2 937 isolates).

Clindamycin resistance was identified in 12.7% (374), and erythromycin resistance was identified in 25.6% (752) of isolates tested. During the study period, a significant increase was observed in the proportion of isolates resistant to each of these antibiotic (for clindamycin 10.5% in 1996 versus 15.0% in 2003, *X* ^2^ for trend = 12.70, *P* < .001, for erythromycin 15.8% in 1996 versus 32.8% in 2003, *X* ^2^ for trend = 55.46, *P* < .001) (see Figure 1). Concurrent resistance to both clindamycin and erythromycin was identified in 12.6% (370) of isolates tested.

**Figure 2:**
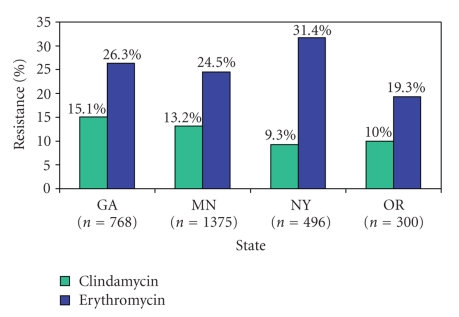
Clindamycin and erythromycin resistance among invasive Group B streptococci isolates by state (*n* = 2 937 isolates).

Significant differences were identified between Minnesota and New York isolates in clindamycin resistance (13.2% versus 9.3%, *P* = .03) and erythromycin resistance (24.5% and 31.4%, *P* < .01). Georgia isolates were more likely to be resistant to clindamycin than New York isolates (15.1% and 9.3%, *P* = .02), but Georgia isolates were less likely to be resistant to erythromycin than to New York isolates (26.3% and 31.4%, *P* = .04) (see Figure 2).

**Figure 3:**
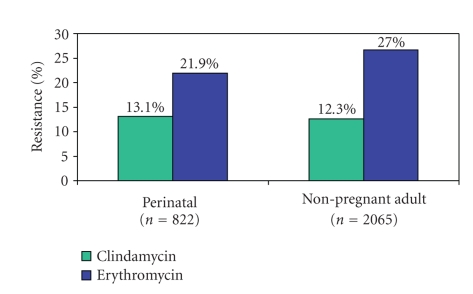
Resistance invasive Group B streptococci isolates among pregnant women and newborns (perinatal) and non-pregnant adults (*n* = 2 887 isolates).

The proportion of isolates resistant to clindamycin and erythromycin did not differ significantly between the perinatal and non-pregnant adult patient groups (see Figure 3). Within the perinatal group, the overall clindamycin resistance was 13.1%, with no significant changes between the early-onset (14.9%), late-onset (10.3%), and maternal populations (13.7%). Likewise, no significant differences in erythromycin resistance levels were identified between the different perinatal groups (21.9% overall; 21.2% for early-onset, 22.1% for late-onset, and 23.9% maternal).

Serotyping was performed on 64.6% (3 471) of isolates. The largest proportion was from Georgia (34.7%), followed by Minnesota (28.5%), New York (23.9%), and Oregon (12.9%). Table 1 illustrates the serotypes identified during the study period, with predominant serotype groups being V, Ia, and III. The proportion of isolates with serotype III was observed to have a significant downward trend (*X* ^2^ trend = 38.8; *P* < .001) during the study period from 25% in 1996 to 17% in 2003; no such trend was observed with the other predominant serotypes.

The most common serotype in the perinatal population was serotype III (37.0%; 351/948). This was significantly different than for non-pregnant adults in which serotype V was most common (31.5%; *P* < .001).

Both susceptibility and serotyping testing were performed on 2 551 isolates. The largest proportion of these isolates were from Minnesota (38.9%), followed by Georgia (30.1%), New York (19.4%), and Oregon (11.8%). Clindamycin resistance was 3.9 times (*P* < .001) more likely to occur in serotype V isolates (25.8%) than in other serotypes (8.2%). In addition, erythromycin resistance was 2.9 times (*P* < .001) more likely to occur in serotype V isolates (42.2%) than in other serotypes (20.2%). Subanalysis within the different disease categories indicated a significant association among clindamycin or erythromycin resistance and early-onset (*P* < .001), late-onset (*P* < .001), and non-pregnant adult populations (*P* < .001). No such association was observed among the maternal population.

Three hundred fifty-five erythromycin-resistant (47%) isolates were further characterized. Seventy-eight isolates (22%) had an M phenotype and all of these isolates contained *mef* only. Fifty-two percent (184/355) were constitutively resistant to clindamycin (cMLS). Seventeen were further tested. Ten were positive for *erm*B and seven contained *erm*TR; one isolate had both *mef* and *erm*TR and one isolate had both *mef* and *erm*B. Twenty-five percent of isolates had inducible clindamycin resistance (iMLS). All of the iMLS isolates were tested for resistance genes; 83 (92%) contained *erm*TR, 1 isolate contained *erm*B, and 6 isolates were negative for *erm*TR and *erm*B.

Univariate analysis was conducted comparing resistance genes to serotypes. Serotype V was associated with a lower proportion of *mef* positive isolates compared to all other serotypes (*P* < .01). In contrast, serotypes Ia, Ia/c, and III were associated with a higher proportion of *mef*-positive isolates compared to all other serotypes (all *P* values <.01). Serotype V had a higher proportion of isolates with *erm*TR, 73% of 37 tested compared with 29% among all other serotypes (*P* < .01).

## 4. CONCLUSIONS

This study demonstrated the prevalence of resistance to antibiotics commonly used for prophylaxis or treatment of GBS infections among a large, multisite, collection of isolates. We found that all invasive GBS isolates tested were susceptible to penicillin and ampicillin, the first-line agents recommended for IAP and to cefazolin and vancomycin, second-line agents recommended for use among IAP candidates who report penicillin allergies. In contrast, GBS resistance to clindamycin (12.7%) and erythromycin (25.6%) was common. Subanalysis of early-onset patient isolates indicated clindamycin resistance at 14.9% and erythromycin resistance at 21.2%. These findings are consistent with previous reports in the literature [3–8] and support the 2002 guidelines requiring susceptibility testing of isolates from IAP candidates with penicillin allergy. Of note, because of reports of subtherapeutic concentrations in amniotic fluid and fetal serum, certain healthcare specialists do not recommend using erythromycin for IAP [15]. Clinicians should be aware of the potential for MLS resistance when determining empiric regimens that target GBS disease.

We found an overall concordance between the MLS phenotype and resistance genotype. D-testing revealed inducible clindamycin resistance in *erm*-containing GBS isolates and also detected resistance in isolates with determinants not identifiable by existing PCR methods. Our findings support the 2002 CDC guideline to test both erythromycin and clindamycin susceptibilities if one of these agents is being considered. Further D-testing should be done if erythromycin resistance and clindamycin susceptibility are found. Rarely, clindamycin resistance without concurrent erythromycin resistance can be observed; this has been associated with the *lin*B gene [9]. Minimal differences were identified in clindamycin and erythromycin resistance levels across the four geographic areas as well as between the perinatal and non-pregnant adult groups. This indicates that GBS-resistance patterns might reflect more of a national than a regional phenomenon.

We noted high cefoxitin MICs to GBS among our GBS isolates. This is not surprising because cefoxitin is a cefomycin, a class of antibiotics which typically provides optimal coverage for anaerobes but lacks adequate activity against gram-positive bacteria. Cefoxitin is likely to provide suboptimal GBS coverage for chorioamnionitis and postpartum endometritis.

The predominant GBS serotypes were Ia, III, and V, which together comprised approximately 70% of all isolates serotyped. The predominance of these serotypes among the perinatal population is consistent with reports in the literature [4, 11] as is the association between serotype V and erythromycin or clindamycin resistance [4, 7]. We found an association of serotype V with the *erm* genotype which confers resistance to both erythromycin and clindamycin.

This study has several limitations, including variability in surveillance periods for isolates collected, a varying proportion of cases with isolate collection and susceptibility and serotyping results, and differences in surveillance populations. However, the ABCs surveillance system is an active, multiregional, and population-based system, and the methods to determine susceptibilities and serotyping were standardized. Continued surveillance of invasive GBS disease including susceptibility and serotype determinations will impact plans for prevention including IAP agents and vaccine design.

## Figures and Tables

**Table 1:** Distribution of serotypes and resistance to erythromycin and clindamycin among invasive group B streptococcus isolates.

		Serotyped isolates with susceptibility results		
Serotype	Serotyped isolates	Total	Erythromycin-resistant	Clindamycin-resistant
	no. (%)*		no. (%)	no. (%)
	(*n* = 3471)	(*n* = 2551)	(*n* = 669)	(*n* = 301)

Ia, Ia/c	835 (24.1)	573	112 (17)	13 (4)
Ib, Ib/c	296 (8.5)	231	37 (6)	10 (3)
II, II/c	357 (10.3)	255	68 (10)	46 (15)
III, III/c	680 (19.6)	496	78 (12)	26 (9)
V, V/c	951 (27.4)	695	293 (44)	166 (55)
NT, NTc	247 (7.1)	175	65 (10)	30 (10)
Other**	105 (3.0)	120	16 (2)	10 (3)

*of isolates serotyped. **IV, IV/c, VI, VI/c, and VIII.
